# Supplementary material for: Non-Hermitian polariton–photon coupling in a perovskite open microcavity
Source: Nanophotonics. 2024 May 15;13(14):2491–500. doi: 10.1515/nanoph-2023-0830 (PMC11636459; doi:10.1515/nanoph-2023-0830)
Supplement: Supplementary file 1 — Supplementary Material Details [file j_nanoph-2023-0830_suppl_001.pdf]

Research Article

M. Kędziora, M. Król, P. Kapuściński, H. Sigurðsson, R. Mazur, W. Piecek, J.Szczytko, M. Matuszewski, A. Opala, and B. Piętka*

Supporting Information: Non-Hermitian polariton-photon coupling in a perovskite open microcavity

1 Crystalisation of PEPI-F

Synthesis of the solution for PEPI-F crystallization was carried out in an argon-filled glovebox by mixing PbI_2 and (4F)-PEAI anhydrous powder in a 1:2 molar ratio. They were then dissolved in γ-butyrolactone so that the perovskite concentration was 0.25 M. The dissolution was carried out for one hour at 50 °C. After the perovskite was removed from the glovebox, 2 μl of PEPI-F solution was used for crystallization and placed between the DBR and oxygen plasma-activated glass. The solution thus sandwiched was sealed in a Teflon jar with 2 milliliters of dichloromethane which served as an antisolvent. After 12 hours, the resulting crystals were dried in a nitrogen flow and the thickness was adjusted by mechanical exfoliation using a layer of PDMS as a tape.

M. Kędziora, P. Kapuściński, J.Szczytko, Institute of Experimental Physics, Faculty of Physics, University of Warsaw, ul. Pasteura 5, PL-02-093 Warsaw, Poland
M. Król, Institute of Experimental Physics, Faculty of Physics, University of Warsaw, ul. Pasteura 5, PL-02-093 Warsaw, Poland; and Research School of Physics, The Australian National University, Canberra, ACT, 2601, Australia
R. Mazur, W. Piecek, Institute of Applied Physics, Military University of Technology, Warsaw, Poland
H. Sigurðsson, Institute of Experimental Physics, Faculty of Physics, University of Warsaw, ul. Pasteura 5, PL-02-093 Warsaw, Poland; and Science Institute, University of Iceland, Dunhagi 3, IS-107, Reykjavik, Iceland
M. Matuszewski, Institute of Physics, Polish Academy of Sciences, Aleja Lotników 32/46, PL-02-668 Warsaw; and Center for Theoretical Physics, Polish Academy of Sciences Aleja Lotników 32/46, 02-668 Warsaw, Poland, E-mail: mmatuszewski@cft.edu.pl
A. Opala, Institute of Experimental Physics, Faculty of Physics, University of Warsaw, ul. Pasteura 5, PL-02-093 Warsaw, Poland; and Institute of Physics, Polish Academy of Sciences, Aleja Lotników 32/46, PL-02-668 Warsaw, Poland, E-mail: aopala@fuw.edu.pl
***Corresponding author: B. Piętka,** Institute of Experimental Physics, Faculty of Physics, University of Warsaw, ul. Pasteura 5, PL-02-093 Warsaw, Poland, E-mail: Barbara.Pietka@fuw.edu.pl

2 AFM measurments

To determine the surface roughness, AFM measurements were taken of the crystal under study. Figure 1 shows a fragment of the crystal with multiple terraces resulting from the layered structure of PEPI-F (top panel) and a fragment of a homogeneous crystal (bottom panel). The calculated RMS of the roughness is 0.46nm

3 Linewidths

In Figure 2, we compare experimental full-width at half maximum (FWHM) with values calculated numerically by the transfer matrix method for two polariton modes of different energies. The results obtained exhibit a reasonably good quantitative and qualitative agreement. Here, the narrowest lines occur near the minimum of the mode, where the polariton-photon branch becomes mostly photonic, as opposed to the broadening of the line observed at the inflection points—indicative of the transition to a more polariton-like state.

4 Air gap simulation

A simulation of how the addition of an air gap affects the dispersion of a perovskite microcavity can be seen in Figure 3 and 4.

5 Fitting and simulation parameters

We performed the fitting dispersion parameters based on the reflection spectrum using the non-linear least

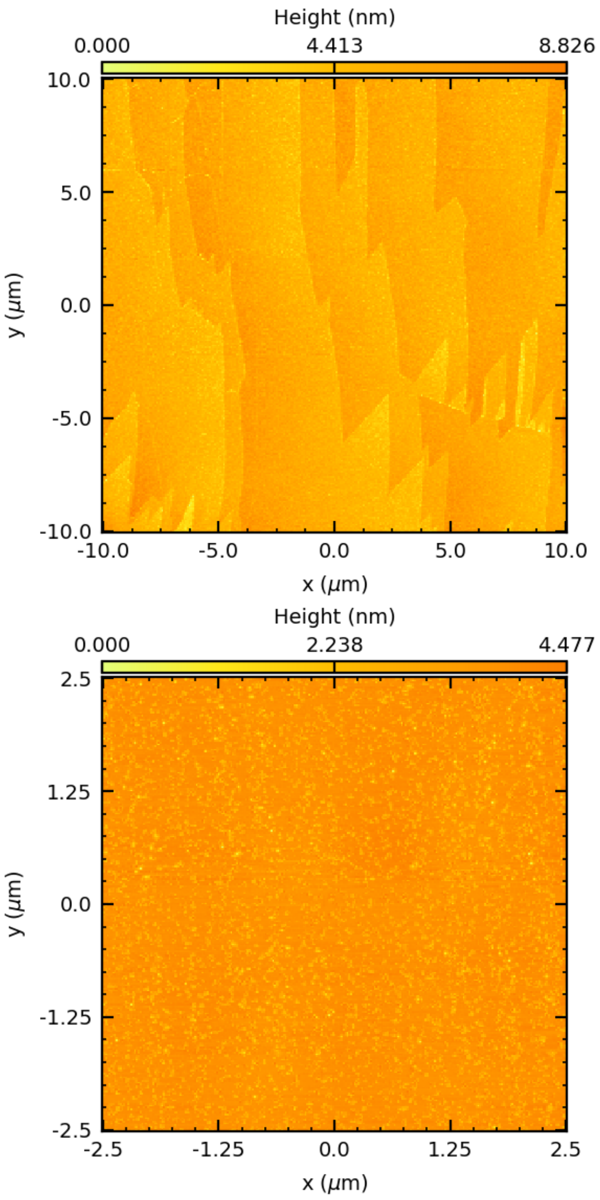

Fig. 1: Atomic force microscope measurements of the test sample. The upper panel shows a fragment with a large number of faults caused by the layered structure of perovskite, the lower panel shows a homogeneous fragment of the crystal surface.

squares method from the SciPy package. The fitting parameters are: $E_{C,0}^{(1)} = 2328.44$ meV, $E_{C,0}^{(2)} = 2440.46$ meV, $E_{C,0}^{(3)} = 2546.79$ meV, $E_{C,0}^{(4)} = 2653.65$ meV, $E_{C,0}^{(5)} = 2764.89$ meV. The rest of the parameters are taken as below: $E_{X,0} = 2389.74$ meV, $\hbar\Omega_R = 329.10$ meV, $\hbar\gamma_C = 0.00658$ meV, $\hbar\gamma_X = 30$ meV, $\hbar\gamma_E = 0.00658$ meV, $m_C^* = 0.0016$, $m_{E,1}^* = 0.18 \cdot 10^{-3}$, $m_{E,2}^* = 0.23 \cdot 10^{-3}$, $m_{E,3}^* = 0.23 \cdot 10^{-3}$. $E_{E,0}$ for CE_1, CE_2 and CE_3 are 1997.30 meV, 2209.60 meV and 2360.50 meV. Parameters $E_{E,0}$ for CE_I and CE_{II} are 2032.08 meV and 2300.0 meV. Here, we assume a small change in the external photon effective mass for different modes to achieve the best agreement with the experimental data. The coupling parameters for photonic fraction are: $J_C^{(1,1)} = 14.49$ meV, $J_C^{(1,2)} = 9.45$ meV, $J_C^{(1,3)} = 8.50$ meV, $J_C^{(1,4)} = 4.79$ meV, $J_C^{(1,5)} = 3.78$ meV. Coupling parameters for excitonic fraction are given by: $J_X^{(1,1)} = 48.20$ meV, $J_X^{(1,2)} = 31.50$ meV, $J_X^{(1,3)} = 28.35$ meV, $J_X^{(1,4)} = 15.96$ meV, $J_X^{(1,4)} = 12.60$ meV. We assume that $J_C^{(n,1)}$ equals $J_C^{(n,2)}$ and $J_X^{(n,1)}$ equals $J_X^{(n,2)}$ to reduce the number of free parameters. The most accurate correspondence between the model and the calculated eigenenergies is achieved by adjusting the J parameters for different n and l values separately. This phenomenon can be attributed to the reduction in strong coupling for higher modes energies. Wavevectors were normalized by taking $k_0 = 11.86\ \mu m^{-1}$. For planar microcavities in-plane wavevector is directly related to incidence/reflectance angle θ through the relation $k_{\|} = \frac{E}{\hbar c} \sin\theta$.

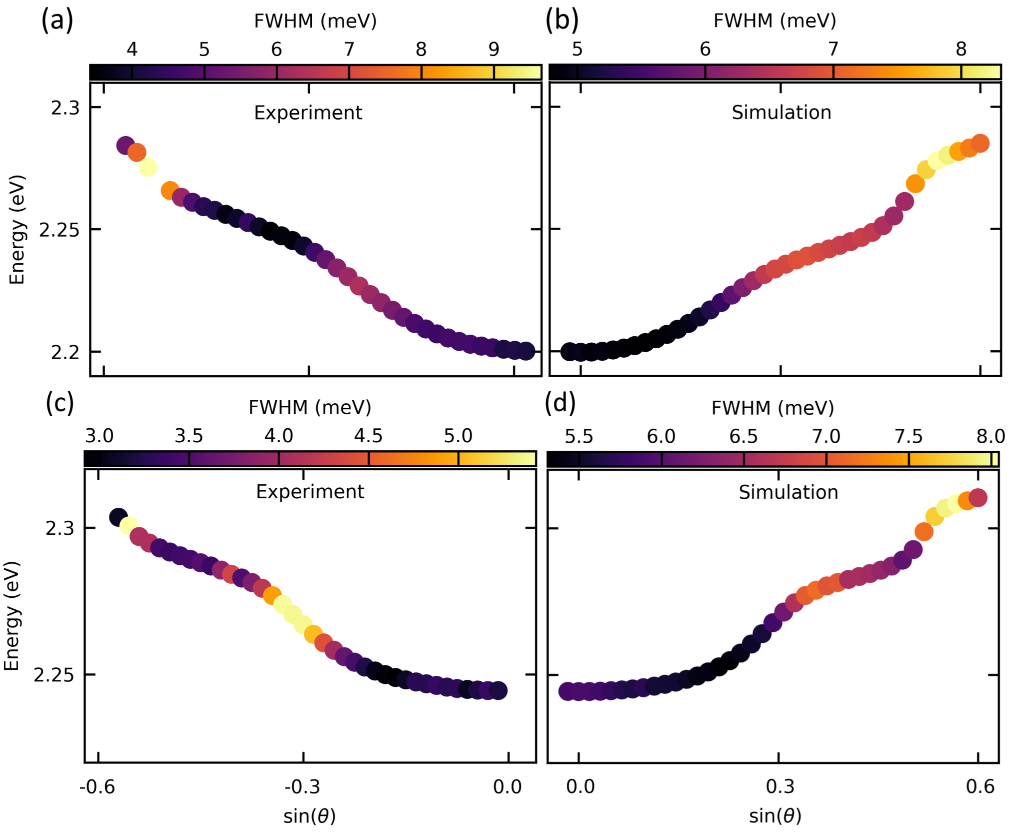

Fig. 2: Comparison of FWHM for experimental results (a,c) and simulations with the transfer matrix method (b,d) from data illustrated in Figure 3a. Panels (a,b) show the mode having a minimum at an energy of about 2.26 eV and (c,d) of about 2.20 eV.

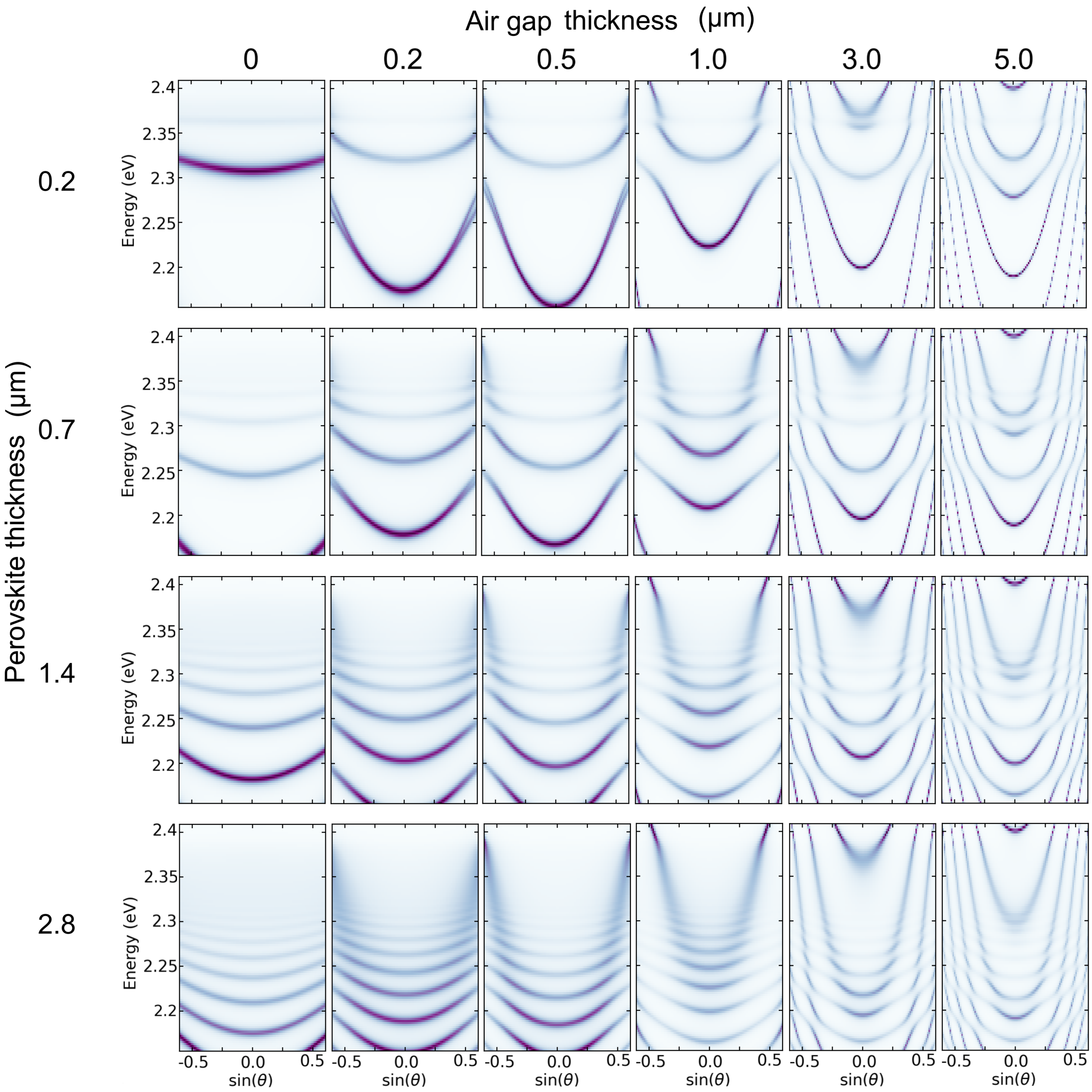

Fig. 3: Simulation of the effect of adding an air layer whose thickness in μm is in the row at the top affects the PEPI-F spectrum of the crystal thickness specified in the column on the left.

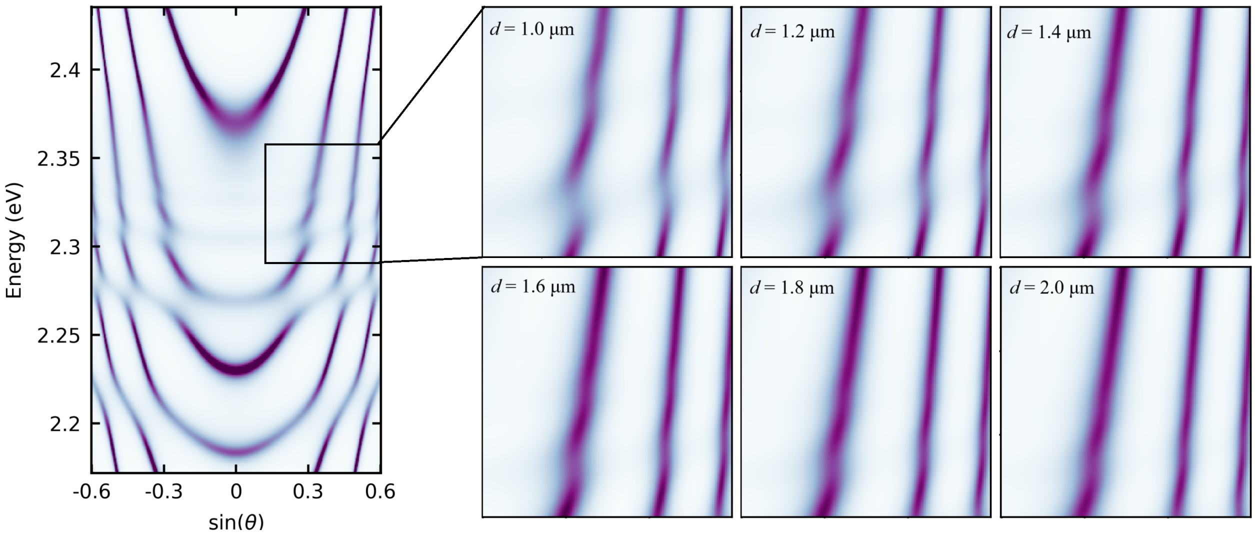

Fig. 4: Representation of closing the energy gap by changing the perovskite thickness in the transfer matrix method.
